# An Evidence-Based Strategic Approach to Prevention and Treatment of Dry Eye Disease, a Modern Global Epidemic

**DOI:** 10.3390/healthcare9010089

**Published:** 2021-01-17

**Authors:** Pragnya R. Donthineni, Swapna S. Shanbhag, Sayan Basu

**Affiliations:** 1 The Cornea Institute, L V Prasad Eye Institute, Hyderabad 500034, India; drpragnyarao@lvpei.org (P.R.D.); swapnashanbhag@lvpei.org (S.S.S.); 2 Center for Ocular Regeneration (CORE), L V Prasad Eye Institute, Hyderabad 500034, India

**Keywords:** dry eye, prevention, levels of prevention, dry eye disease, tiers of prevention, computer vision syndrome

## Abstract

Dry eye disease (DED) is an emerging health concern causing significant visual, psychological, social, and economic impact globally. In contrast to visual rehabilitation undertaken at late stages of DED, measures instituted to prevent its onset, establishment, or progression can alter its natural course and effectively bring down the associated morbidity. This review attempts to present the available literature on preventive strategies of DED at one place, including strategies for risk assessment and mitigation, targeting a wide range of population. A literature search was conducted using PubMed and an extensive literature review on preventive strategies for DED was compiled to put forth a holistic and strategic approach for preventing DED. This can be undertaken at various stages or severity of DED directed at different tiers of the health care system. Conclusion: This review intends to put emphasis on preventive strategies being adopted as an integral part of routine clinical practice by general ophthalmologists and specialists to tackle the burden of DED and improve the quality of the lives of the patients suffering from it.

## 1. Introduction

Dry eye disease (DED) is a multifactorial disease caused by a vicious cycle of dysregulated ocular inflammation leading to chronic ocular surface dysfunction [1]. The prevalence of DED has been increasing globally among all age groups, with escalating rates being reported among children and adolescents [2,3]. The emergence of a digital revolution and increasing dependence on video display units (VDUs) further elevates the risk of DED [4]. While patients with pre-existing disease continue to suffer owing to its chronic clinical course, the addition of new cases with time will only increase the overall disease burden. The end-stage ocular surface disease resulting from chronic and advanced DED is exceptionally challenging to manage and has substantial cost implications [5]. Apart from causing significant ocular morbidity, DED can commonly be associated with underlying systemic autoimmune pathologies that could potentially be life-threatening. These patients also experience role limitation, pain, and poor general health, significantly impacting their quality of life (QoL) comparable to the QoL for serious debilitating medical illnesses such as chronic kidney disease and severe angina [6]. Additionally, DED has also been associated with anxiety, depression, and sleep disorders and can have a great deal of economic, social, and psychological impact on the individual suffering from it [6,7]. 

With the ongoing epidemiological transition, there is a shift in disease patterns from communicable illnesses such as infections to the dominance of non-communicable chronic diseases. Recent epidemiological studies have estimated that the annual incidence and prevalence of DED would continue to rise significantly in the coming years [8,9]. This becomes even more pertinent during situations such as the global COVID-19 pandemic, where person-to-person interactions are being minimized at the cost of increased dependence on technology and VDUs. Thus, the belief that “an ounce of prevention is worth a pound of cure” is extremely relevant in the current times. It is, therefore, essential for treating physicians to acknowledge that the effect of treatment in controlling a disease is unsustainable without having appropriate preventive strategies in place. Thus, while we continue to focus on effectively treating patients with DED, it is crucial to direct efforts towards establishing preventive strategies to bring down the overall impact of the disease. These include primordial prevention intercepting emergence of risk factors, primary prevention to prevent the onset of the disease, secondary prevention aiming at early diagnosis and treatment and tertiary prevention to limit complications and visually rehabilitate patients with DED. Thus, in this review, the authors aimed to discuss an integrative approach to manage DED that emphasizes on preventive and promotive strategies at various levels of health care.

## 2. Methods and Literature Review

In August 2020, the literature searches pertaining to the components of this review were completed using PubMed. The keywords used for the study were “dry eye,” “dry eye disease,” “computer vision syndrome,” “digital eye strain,” “video terminal users,” “office ergonomics training,” and “prevention of dry eye.” As the volume of literature recovered was huge, we included only articles in the English language that had relevance with primordial, primary, secondary, tertiary, or quaternary prevention of DED. This significantly narrowed down our search, and the final articles were included for the current literature review. 

## 3. Levels of Prevention of DED

**Figure 1:**
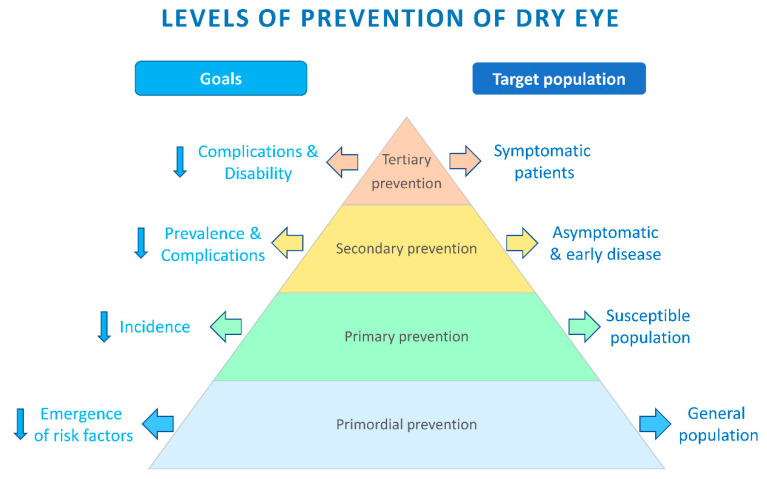
Tiers of prevention of dry eye disease (DED): this figure describes the pyramid depicting the preventive strategies at various levels of health care with the goals (arrows on the left) and the target population (arrows on the right).

One of the best aspects of healthcare reforms is the emphasis laid on the prevention of disease. While we clinicians strive to excel in treating a manifested illness, there is a pressing need to advance equally the prevention of its establishment and progression. In order to achieve this, measures should be directed at various tiers of prevention (Figure 1) [10]. Like any other disease, the strategies to prevent DED can be classified into the following five levels:

1. Primordial prevention;
2. Primary prevention;
3. Secondary prevention;
4. Tertiary prevention;
5. Quaternary prevention.

### 3.1. Primordial Prevention of DED

This tier of prevention includes measures that can avoid the emergence and establishment of social, cultural, and economic patterns of living that can elevate the risk of DED [10]. Thus, for primordial prevention, we need to target the general population as a whole to prevent the emergence of risk factors of DED.

#### 3.1.1. Strategies for Primordial Prevention

##### Epidemiological Research

Epidemiological research is of paramount importance to identify the various risk factors of DED. Identifying underlying conditions that can lead to exposure to causative factors can go a long way in addressing this disease at its grassroots. Numerous epidemiological studies have reported age, sex, place of residence, urbanization, occupation, and socio-economic status as significant risk factors for developing DED [9]. Increased risk of DED has also been associated with prolonged exposure to sunlight, indoor smoke, and smoking [11]. With growing urbanization and digitization, along with dynamically changing environmental exposures, we can anticipate a greater surge in cases of DED in the coming years. Epidemiological research can thus help healthcare agencies in policymaking to establish comprehensive public healthcare measures to tackle this emerging disease.

##### Lifestyle Modifications

Lifestyle changes such as increased use of VDU’s, sedentary lifestyle, and a diet deficient in Vitamin A, Vitamin D, or Omega-3 fatty acids could contribute to the emergence of DED [12,13,14]. Vitamin A deficiency continues to significantly cause DED among children in developing countries, despite being preventable [15]. The resulting dryness in Vitamin A deficiency is due to either lacrimal insufficiency or poor surface wettability caused by altered glycocalyx and reduced goblet cells [16]. Vitamin D is known to decrease ocular surface inflammation and improve the tear film osmolarity, stability, and corneal epithelial barrier function [17]. Hwang et al. reported that Vitamin D improved the efficacy of lipid-based topical lubricants when it was administered as adjuvant therapy in patients with DED [18]. This could be due to the augmented production of ocular surface surfactants that enhance the miscibility of lipid-based formulations into the aqueous layer of the tear film. Thus, dietary supplementation with Vitamin A and D and behavioral changes such as a reduction in screen time and increased physical activity can help modify health-damaging exposures if promoted from childhood [19,20]. A few short-term studies reported a positive role of Omega-3 fatty acid supplementation in treating DED [21,22]. However, the dry eye assessment and management (DREAM) study revealed that Omega-3 fatty acid supplementation offered no additional long-term benefit over placebo in alleviating the symptoms. Its extension withdrawal study also reported results consistent with the former study [23,24].

##### Health Education

Health education promoting ocular surface protective measures can be undertaken among the general population, especially targeting children, adolescents, and other high-risk groups [3,25]. Conducting workshops to impart skill transfer to modify lifestyle and workplace ergonomics can be beneficial. Utilizing mass media for health education in the community can help create a conducive ecosystem where health-related measures could become a new normal [25].

##### Genetic Screening

DED can also be caused by rare inherited diseases, such as anhidrotic ectodermal dysplasia, epidermolysis bullosa, Riley Day syndrome, Ichthyosis, and congenital alacrimia, and their associations, such as Allgrove or Triple-A syndrome, lacrimal-auriculo-dento-digital (LADD) syndrome, and Pierre Robin sequence. Genetic screening of the population at risk, though expensive, can prove to be helpful in such scenarios [17].

##### Legislative Regulations and National Policies

Most countries across the globe have work related regulations governed by legislature, to improve productivity while ensuring safety of the working class. Thus, having work hour restrictions for individuals exposed to prolonged screen time can help reduce the risk of DED in this specific group. Occupational exposure to chemicals and industrial accidents can also cause DED [26]. The risk of exposure to such occupational hazards can be mitigated by having mandatory protective measures for individuals at high risk for ocular chemical, thermal, or radiation injuries. Environmental pollution and particulate matter exposure have also been associated with DED and can be effectively addressed only when the countermeasures are planned as legislative policies to conserve the environment [11,27]. 

### 3.2. Primary Prevention

This level of prevention includes measures taken to prevent the onset of disease by controlling the causes and risk factors and targeting the susceptible population. Interventions can be applied before there is any evidence of disease to bring down the overall incidence of DED [10]. This can be achieved by using population strategy, individual high-risk strategy, or a combination of both. In population strategy, attention is focused on the entire population to reduce the average risk of disease, whereas in the individual high-risk strategy, individuals vulnerable to disease-specific exposure are targeted [10].

#### 3.2.1. Strategies for Primary Prevention

##### Screening for the Presence of Risk Factors

Identifying risk factors for developing DED among individuals is the most crucial aspect of this tier of prevention. Screening the general population for risk factors for DED with a particular focus on high-risk groups, such as those in the geriatric age group, perimenopausal women, contact lens users, or those with long-term use of systemic and topical medications, can be helpful [28]. Common ocular conditions, such as meibomitis and demodex blepharitis have been associated with developing DED, which need to be looked for in all patients presenting to the clinics [17]. Individuals with increased screen time, such as software professionals and adolescents, are also at high risk for developing DED [12]. Prolonged exposure to VDUs is associated with a reduction in the blink rate by 40–60% and an incomplete blink amplitude as compared to normal individuals [29,30,31]. This could contribute to the rapid desiccation of the tear film, causing ocular fatigue and subsequent development of computer vision syndrome (CVS) [32]. 

##### Minimizing Iatrogenic DED

Iatrogenic dry eye disease is known to be caused by contact lens wear, long term use of topical and systemic medications, and interventions such as corneal refractive surgery, keratoplasty, cataract, and lid-related surgeries [28]. Various medications with anticholinergic activity, such as anti-depressants, neuroleptics, antihistamines, and anti-hypertensives, have been associated with DED [28]. This action is attributed to the involvement of G-protein coupled M (muscarinic) receptors in the lacrimal gland acini and conjunctival goblet cells, leading to aqueous and mucin deficiency and subsequent DED [28]. On the other hand, adrenergic agonists, such as beta-blockers and alpha agonists, can also alter tear film volume and quality via the protein kinase C pathway [33]. Thus, once an iatrogenic risk factor is identified by screening, various strategies for risk mitigation can be undertaken to prevent the onset of DED in the future. 

##### Health Education

Imparting education to increase the knowledge and awareness of early signs and symptoms of DED among high-risk groups can be instrumental in disease prevention. Enabling these patients to detect symptoms early by themselves during the disease course can translate into effective preventive practice. Numerous crowd-sourced cross-sectional studies using smartphone applications have identified individuals with undiagnosed and diagnosed DED and its associations, such as clinical depression [34]. Additionally, training individuals with prolonged VDU to adopt office ergonomic practices has helped improve visual symptoms and increase work productivity [35]. 

##### Workplace Ergonomics and Modifications

The work environment can play an essential role in developing and perpetuating the vicious cycle of DED. Factors such as low humidity, air pollution, screen brightness, and ambient light among VDU users have been associated with the risk of developing DED and reduced work performance [36]. Thus, appropriate work environment modifications can alleviate these negative influences and increase work performance. Recommended improvements include an appropriate location of the screens, with eyes looking downward by 15–20 degrees and 60–100 cm away while working [37,38]. Other recommendations include reducing the level of screen brightness by using screen guards and blue filters [35], reducing glare from ambient light by using blinds and screens over windows and light sources [28], and maintaining a proper body posture with well-adjusted chair height and wrist support while typing [32].

##### Eye Protective Health Practices during VDU Use

Measures protecting ocular health while using VDUs can include refractive error correction, taking frequent breaks once every 20–30 min, preferably by looking at a distant object [39], blinking at regular intervals, and using protective eyewear with anti-glare tinting [40]. Various software applications facilitating users to set reminders that prompt them to blink and stretch the body at regular intervals have reported being useful [41].

##### Micronutrients and Nutraceutical Supplementation

Supplementation of micronutrients and nutraceuticals can prove to be useful in individuals susceptible to developing DED [42]. This can be achieved either by recommending dietary changes or by prescribing nutraceuticals. Dietary nutraceuticals are concentrated or the purified form of the micronutrients, such as antioxidants, omega-3 fatty acids, and multivitamins, are extracted from food products and administered as medicinal formulations. Antioxidant complex supplementation (including beta-carotene, ascorbic acid, Vitamin B, E, Zinc, and copper), when administered in a crossover placebo-controlled, double-masked randomized control trial, has demonstrated improvement of tear film stability, squamous metaplasia, and goblet cell density without significant change in tear volume [43]. Though some benefit has been demonstrated in the literature, more research needs to be carried out to look at the accurate composition, dosage, and indications of nutraceutical supplementation [43,44].

### 3.3. Secondary Prevention

This level of prevention aims at early detection and early treatment of DED. Interventions are more effective in the early stages where there is a window of opportunity to either reverse the disease or halt its progression. Thus, targeting patients with asymptomatic and early disease can reduce the prevalence of DED and prevent the morbidity associated with it.

#### 3.3.1. Strategies for Secondary Prevention

##### Screening for the Presence of DED

Recognizing the presence of risk factors among patients and having a low index of suspicion can help identify asymptomatic patients in the early stage of DED. Screening the patients at risk, such as those with meibomitis, diabetes, autoimmune diseases, contact lens use, prolonged VDT use, or long-term use of topical or systemic medications associated with DED, can help with early diagnosis. Tear film osmolarity is superior to questionnaires like ocular surface disease index (OSDI )as a more reliable screening tool to detect ocular surface alterations, as studied in individuals with prolonged VDT use [45]. 

##### General Health Measures

Health measures, such as whole-body hydration, lid scrubs, lid massage, warm compresses, and use of topical lubricants, are measures that can easily be followed by all patients of DED [46]. The use of eye guards and humidifiers at home and in the workplace can prevent disease progression among office workers [39,47,48]. Isolation of the ocular surface by using microenvironment glasses (MEGS) in combination with the use of artificial tears and environmental manipulations is reported to be most effective in treating patients with CVS. Moist, cool air devices have also been reported to alleviate dry eye symptoms and improve tear film stability, especially among prolonged VDT users [49].

##### Medical Therapy

The options for medical therapy of DED are vast beyond just the use of topical lubricants, which is considered the traditional line of management. Numerous formulations of preservative-free artificial tears are available with variable combinations of aqueous base and viscosity enhancers that are safe and effective in treating mild to moderate DED [50]. Apart from lubricants, topical treatment with either 0.5% Loteprednol etabonate or 1% Methylprednisolone for induction and maintenance with topical cyclosporine provided faster symptomatic relief clinical improvement of the ocular surface that can limit associated keratopathy [51,52]. Newer osmoprotectants, such as Trehalose, can protect the ocular surface from desiccation, apoptosis, and oxidative stress and maintain corneal epithelial homeostasis, which could be invaluable if initiated in the early stages of DED [53]. Lipid-based preparations or emulsions have been gaining popularity and are reported to be useful, especially in MGD and lipid layer deficiency, as they address the root cause of poor lipid layer quality [54]. Other newer drugs, such as topical secretagogues, are also being explored in the treatment of DED. Diquafosol tetrasodium, a purinergic P2Y2 receptor agonist, is one such drug that has been reported to stimulate mucin from conjunctival epithelium and goblet cells [55]. Another mucin secretagogue, Rebamipide, has shown promising results with improvement in subjective symptoms, tear film breakup time (TBUT), lissamine conjunctival staining, and corneal fluorescein staining following therapy [56]. Furthermore, it has also been effective in treating lid wiper epitheliopathy and short TBUT DED [57]. Lifitegrast, a newer integrin antagonist, has shown a reduction in T cell migration, recruitment, and cytokine release having a potential application in therapy of DED [58]. Timely and prompt initiation of appropriate treatment in the early stages of DED, targeting inflammation and component deficiency, can limit keratopathy and minimize complications.

##### Surgical Management

Though surgical management is not always required in asymptomatic and early stages of DED, some minor procedures have been found to be beneficial in limiting ongoing damage due to the chronic course of DED. Enhancement of aqueous retention by techniques causing temporary or permanent punctal occlusion can help improve the symptoms of DED. This could be achieved by either placing absorbable or non-absorbable punctal plugs or by surgical punctal occlusion [59]. Surgical punctal occlusion can generally be achieved by using partial or total thermal punctal cauterization, sutured punctal pugs [59], conjunctival flap or graft placement over the punctum [60], extirpation of the canaliculus, or canalicular ligation [61]. These procedures come with their own set of risks and benefits and need to be performed judiciously only when indicated [59].

### 3.4. Tertiary Prevention

In this level of prevention, interventions are designed to arrest the progression of an established disease, and thereby, control the complications among symptomatic patients [10]. The primary aim is to minimize the associated disability and suffering. This is the most taxing among all the preventive tiers of the health care system due to the long-term management of chronic disease involved and the substantial cost of surgery and rehabilitation [5]. Though tertiary prevention incurs higher costs while catering to smaller populations, most ophthalmologists, unfortunately, limit their preventive practice to only this level of prevention. 

#### 3.4.1. Measures for Tertiary Prevention

##### Diagnosis of Underlying Systemic Associations

DED is commonly associated with underlying systemic diseases, such as connective tissue disorders and autoimmune bullous diseases, that are often both sight- and life-threatening [62]. Thus, a detailed systematic work-up to rule out both ocular and systemic associations is mandatory to prevent progression in every established case of aqueous deficiency DED [62]. An algorithmic approach, including a detailed history, clinical examination, serological evaluation, and additional specific and ancillary investigations, are required to unmask the underlying systemic associations in these patients [63]. 

##### Medical Therapy

Medical therapy, including ocular with or without systemic immunosuppressive therapy (IMT), in addition to supportive measures, such as ocular lubricants, are generally useful based on the etiology of DED [46]. Temporally limited causes of DED, such as Stevens-Johnson’s syndrome (SJS) and chemical/thermal burns, are non-progressive, and the sequelae depend on the extent of damage occurring during the one-time insult during the acute phase. Amniotic membrane transplantation (AMT) with adequate medical therapy in the acute stage of SJS can hasten recovery and minimize sight-threatening complications of DED [64]. Likewise, timely and appropriate management of acute chemical burns with medical therapy and AMT can help limit associated ocular morbidity [65]. In progressive causes of DED generally seen with autoimmune pathologies, aggressive intensive IMT in acute phases followed by long term maintenance therapy can help limit disease progression. In addition to medical therapy, the use of scleral lenses such as prosthetic replacement of ocular surface ecosystem (PROSE) lenses helps relieve the symptoms, stabilizing the surface and visual rehabilitation of these patients [66]. 

##### Surgical Management

Some patients may have significant ocular morbidity that would need surgical interventions to either prevent or manage complications and for visual rehabilitation. Cataract surgery is safe and beneficial in patients with DED across various etiologies [67]. In the presence of a visually significant cataract, it is preferable to plan a cataract surgery as soon as possible, before significant keratopathy sets in. Corneal transplantation is not a preferred option for visual rehabilitation due to poor graft survival rates in these eyes with a hostile ocular surface environment [68,69]. Hence, it should be considered a last resort for tectonic stabilization of the globe if required and not for visual rehabilitation. Other techniques for tectonic stabilization of the globe, such as Tenon’s patch graft, multi-layered AMT, cyanoacrylate tissue adhesive application, or corneal patch graft, should be preferred over tectonic penetrating keratoplasty when feasible [70,71,72]. Specialized surgeries, such as limbal stem cell transplantation [73] and Boston type I keratoprosthesis [74], can be undertaken for relatively wet eyes for visual rehabilitation. However, Boston type II keratoprosthesis (Kpro) [75], LVP Kpro [76], or modified osteo-odonto keratoprosthesis [77] are needed in very severe DED with end-stage ocular surface disease.

A combination of medical and surgical interventions followed by scleral lenses are generally needed in advanced cases of DED. Such multimodal approaches have been reported to change the natural course of the disease and limit ocular morbidity in cases with DED related to SJS [78,79].

##### Health Education

**Figure 2:**
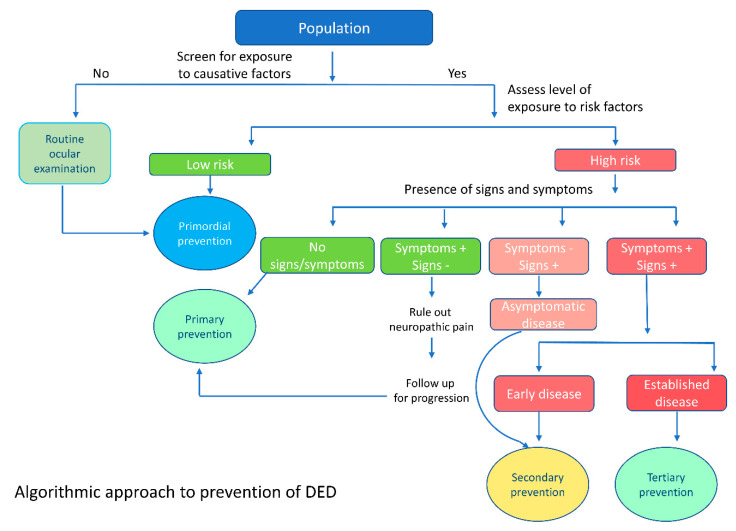
Algorithm for the prevention of dry eye disease: the figure depicts the approach to evaluating a patient with dry eye disease and the institution of various preventive strategies based on the clinical course and disease severity. The low-risk and high-risk stratification is based on the strength of the evidence present in the literature with regards to the association of a risk factor with DED, as stated in the Tear film and ocular surface society – dry eye workshop II (TFOS DEWS II) epidemiology report [2]. High risk includes risk factors such as age, female gender, connective tissue disorders, Sjogrens syndrome, VDU use, contact lens use, Bone marrow transplantation, and environmental factors. Low risk factors include post refractive surgery, allergic conjunctivitis, thyroid disease, psychiatric conditions, diabetes, and low fatty acid intake.

**Figure 3:**
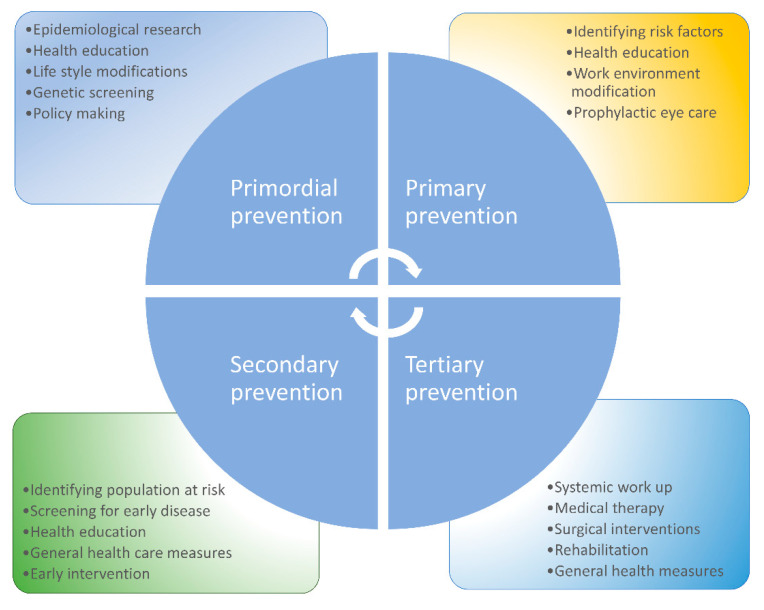
Summary of the various strategies for prevention of dry eye disease: this figure describes the numerous strategies for the prevention of DED at various levels of healthcare.

It is essential to increase awareness among patients, medical and paramedical professionals, and general ophthalmologists about the potential visual rehabilitative measures available to improve the quality of life of patients with end-stage ocular surface disease. Most patients with advanced disease who have a potential for visual rehabilitation end up leading disability-adjusted life years (DALY) due to a lack of awareness or appropriate referral to centers offering specialized and advanced care for ocular surface disease. Advanced ocular surface surgical techniques, such as stem cell transplantation and keratoprosthesis surgery, can help visually rehabilitate these patients [72,74,75]. Apart from treating ocular ailments, consultation and counselling from mental health professionals is vital to deliver holistic care to these patients and effectively handle associated depression, anxiety, and sleep-related disorders [80]. Research has shown that subjective happiness was associated with lower reporting of symptoms of DED, despite the presence of objective signs and vice versa, highlighting the importance of positive psychological interventions in these patients [81]. An algorithmic approach to prevention of DED is described in Figure 2, and a summary of various measures of prevention is described in Figure 3.

### 3.5. Quaternary Prevention

This is a higher level of prevention where the action is taken to identify patients at risk for over-medicalization [81]. The primary objective of this level of prevention is to suggest ethically acceptable interventions to patients and protect them from new medical invasion. It targets the health care system as a whole, intending to reduce iatrogenesis and disease mongering.

#### Strategies for Quaternary Prevention

DED can be caused iatrogenically while managing various ocular and systemic medical conditions related primarily to drug toxicity [28,82]. Additionally, over the counter availability of lubricants with preservatives at lower cost can lead to their long term, widespread use and subsequent ocular surface toxicity [50,83,84]. To ensure a dynamic balance between the risks and benefits of various treatment modalities offered for DED, it is essential to lay down strict technical criteria for medical interventions. Ethical requirements should be laid down carefully, and strict institutional control methods should be put in place in all health care organizations [82]. Systematization of medical care of patients and educating all health care workers on these protective measures can help protect citizens and patients from over-medicalization. It is vital for optometrists and general practitioners, who are the first-line healthcare personnel, to be aware of the entire spectrum of DED and the management strategies available [85,86]. Moreover, public policies and financing should be carried out to ensure compliance and uniformity across the country.

## 4. Conclusions

DED is an emerging disease with a significant economic and humanistic burden, and needs a strategic approach focusing on preventive measures to effectively tackle it. It is imperative for occupational physicians, general practitioners, general ophthalmologists, cornea, and ocular surface specialists to undertake risk factor management in addition to delivering conventional care to all their patients. Thus, the first step towards this would be undertaking risk assessment, i.e., watching out for the presence of risk factors of DED in all patients visiting our clinics irrespective of the presenting complaint. After assessing the risk, we need to institute appropriate preventive measures to effectively control the emergence, establishment, and progression of DED. With the millennial technological revolution and lifestyle trends accentuating the evolution of DED as a global health burden, a perfect synergy between curative and preventive measures is the only way to ameliorate the massive suffering associated with this disease.

## Data Availability

Not applicable.
